# The Role of Contrast Adaptation in Saccadic Suppression in Humans

**DOI:** 10.1371/journal.pone.0086542

**Published:** 2014-01-22

**Authors:** Xiao-Jing Gu, Ming Hu, Bing Li, Xin-Tian Hu

**Affiliations:** 1 State Key Laboratory of Brain and Cognitive Science, Institute of Biophysics, Chinese Academy of Sciences, Beijing, China; 2 Key Laboratory of Animal Models and Human Disease Mechanisms, Kunming Institute of Zoology, Chinese Academy of Science, Kunming, Yunnan, China; 3 Department of Neurobiology and Anatomy, University of Texas–Houston Medical School, Houston, Texas, United States of America; 4 University of Chinese Academy of Sciences, Beijing, China; Istituto di Neuroscienze, Italy

## Abstract

The idea of retinal and ex-retinal sources of saccadic suppression has long been established in previous studies. However, how they are implemented in local circuit remains unknown. Researchers have suggested that saccadic suppression was probably achieved by contrast gain control, but this possibility has never been directly tested. In this study, we manipulated contrast gain control by contrast-adapting observers with sinusoidal gratings of different contrasts. Presaccadic and fixational contrast thresholds were measured and compared to give estimates of saccadic suppression at different adaptation states. Our results reconfirmed the selective saccadic suppression in achromatic condition, and further showed that, achromatic saccadic suppression diminished as contrast adaptation was accentuated, whereas no significant chromatic saccadic suppression was induced by greater contrast adaptation. Our data provided evidence for the involvement of contrast gain control in saccadic suppression in achromatic channel. We also discussed how the negative correlation between contrast adaptation and saccadic suppression could be interpreted with contrast gain control.

## Introduction

Saccade is the fast switch between fixation points. Human and non-human primates direct their attention by making frequent saccadic eye movements. In fixation, high visual acuity becomes possible when image of the interested target falls onto the fovea of the retina. However, during and outlasting the saccade execution, substantial decrease of visual sensitivity, known as saccadic suppression, is observed. Saccadic suppression eliminates the drastic visual disturbance elicited by saccade itself, making it possible to acquire a stable percept. Saccade suppression generally starts from an average of 50 ms before saccade, peaks at saccade onset, diminishes all through the saccade duration and finally vanishes about 50 ms after saccade termination [1]. Retinal sources, such as motion blur and visual masking, have been confirmed playing major roles in the visual suppression [2], [3]. However, researchers noticed that retinal contributions were not sufficient to explain all the effects [4]. With the same visual inputs, observers showed different suppression patterns under simulated saccade and real saccade, affirming the necessity of an extra-retinal source of saccadic suppression [1].

Electrophysiological studies have found fluctuations of various significance in different stages of the visual system produced by saccade-visual interaction [5], [6], [7], [8], [9], [10]. However, how saccadic suppression is implemented in local neural circuit has rarely been reported. Results from several psychophysical researches suggest that contrast gain control could be the possible mechanism underlying saccadic suppression. One study utilizing masking paradigm shows that saccade attenuated visual sensitivity at all mask levels by a constant factor, which is a divisive way of gain modulation typically demonstrated in contrast gain control [11]. In another study, impulse response peaks faster during saccade than that in fixation for luminance modulation, consistent with the suggestion that saccadic suppression is mediated by contrast gain control [12]. Moreover, gain control mechanism has been incorporated into computation models to test and explain assumptions of saccadic suppression mechanism [1], [13], [14]. Thus contrast gain control has been raised as an explanation for the above studies, yet no psychophysical experiment is conducted to directly explore the relationship between saccadic suppression and contrast gain control.

Here we propose that saccadic suppression involves contrast gain control. To verify this, we manipulate the contrast adaptation level of participants and measure the contrast threshold under fixation and saccade. By comparing the two contrast thresholds, we derive direct evidence for the influence of contrast adaptation on saccadic suppression and further explain the interaction with contrast gain.

## Methods

### Ethics Statement

This study was conducted according to the principles expressed in the Declaration of Helsinki and had approval from the Human Research Ethics Committee of the Institute of Biophysics, Chinese Academy of Sciences. All participants provided written informed consent for the participation.

### Participants

One female and three males aged from 27 to 32 took part in the experiment. They all had normal or corrected-to-normal vision and normal color perception. Participants were not told about the purpose of the study until the experiment was finished, except for one author (GXJ). All of them had practiced enough trials before final data acquisition so that they fully understood and mastered the techniques required to finish the task.

### Stimulus

**Figure 1:**
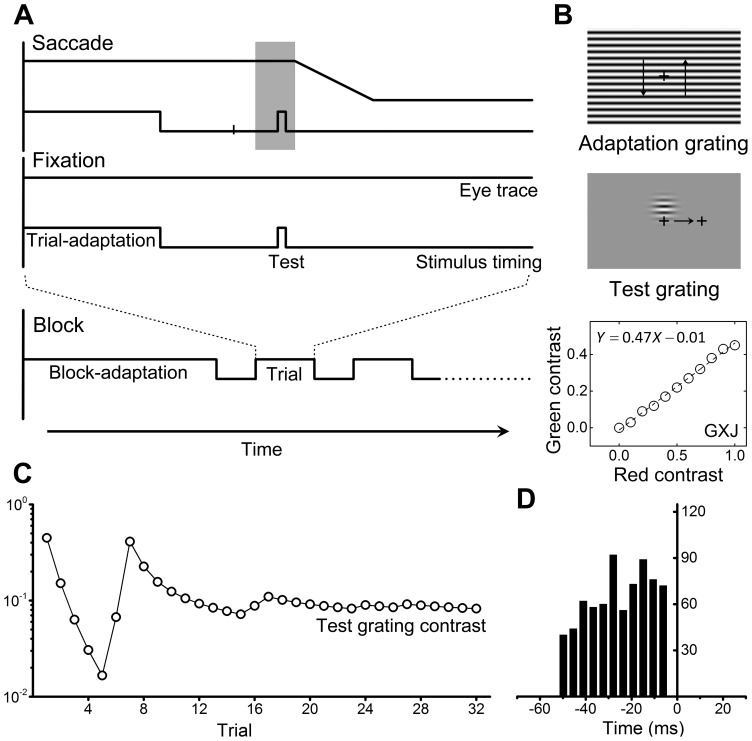
Methods. (A) Schematic illustration of block and trial designs. In saccade trials, the short vertical bar on stimulus timing represents the onset of saccade target, followed by a time interval (gray band) when test grating was presented. In fixation trials, test grating was presented after a comparable interval after adaptation as that of saccade trials. Events are not sequenced with real time scale. (B) Adaptation grating (upper panel) and test grating (middle panel) were used under saccade and fixation (only stimuli of achromatic condition were shown in the figure). The arrows in adaptation grating show the direction of motion, while the arrow in test grating shows the direction of saccade. The lower panel is the equiluminance data of one observer (GXJ) derived from flicker photometry. Dash line denotes the linear fit of the data. Thus the amplitude ratio between green and red channel for this observer is 0.47. (C) Contrast of test grating converges in 32 trials of a single task under the control of QUEST algorithm and a 75% correct threshold is then suggested. (D) Distribution of the time of test grating presentation prior to saccade onset, collected from all participants. Data used here actually ranged from −51 to −5 ms because offline analysis of eye movements uses slightly different saccade velocity criteria from that of online parser. Experiment details are fully described in section **Methods.**

Vertically modulated sinusoidal grating with a spatial frequency of 0.5 cycles/° was used as the carrier to construct the stimuli for both adaptation operation and contrast threshold measurement. A full screen adaptation grating, which was the carrier drifting up and down alternatively with a temporal frequency of 8 (achromatic grating) or 4/3 (chromatic grating) cycles/s, adapted the observers. Drifting adaptation grating was used to avoid local luminance/color afterimage. A static test grating, which was the same carrier enveloped by a 2-D circularly symmetric Gaussian window with a spatial constant of 3°, tested the contrast threshold of observers. The test grating patch was centered randomly above or below the center of the screen with a 4° displacement. Adaptation and test stimuli were illustrated in **Figure 1B**.

The contrast of sinusoidal modulation was defined by Michelson contrast. Achromatic grating (black–white) was composed by a single sinusoidal modulation across red, green and blue channels. Chromatic grating (red–green) was composed by two sinusoidal modulations in red and green channels with a 180° phase shift. The equiluminance of chromatic grating was determined by flicker photometry [15]. An amplitude ratio between red (CIE coordinates: x = 0.652, y = 0.336) and green (CIE coordinates: x = 0.279, y = 0.616) channel was provided by linear-fitting equiluminance data (green contrast versus red contrast) for each observer (**Figure 1B** **lower panel**) to minimize perceived flicker when the chromatic grating was reversed in phase at 20 Hz. After the two amplitudes were associated, for convenience, the contrast of red grating was used as the color contrast measurement of chromatic modulation, instead of the RMS (Root Mean Square) cone contrast commonly defined in color vision. The contrasts of achromatic and chromatic grating were dynamically controlled by QUEST procedures respectively [16]. Three adaptation contrasts were used: 0, 0.2 and 0.85. For participant TH, lower adaptation contrasts, 0, 0.1 and 0.43 were used in chromatic experiment to avoid ceiling effect. The contrast combinations were composed with approximately equal-spaced values on logarithmic scale (we say “approximately” here because the lowest contrast was replaced by zero contrast since it was hardly discernable).

Stimulus was presented on a 22″ LCD monitor (SAMSUNG 2233RZ) [17] at a refresh rate of 120 Hz, with a gray scale of 8 bits/pixel and a spatial resolution of 1680×1050 pixels, subtending a visual area of 45°×30° at the distance of 57 cm. The monitor was calibrated with Spyder 3 Elite (Datacolor) so that display luminance was linear with gray scale value. LCD backlight was then adjusted to achieve an average luminance of 52 cd/m^2^. The display was turned on 30 min prior to every experiment session to stabilize the luminance level. Participants ran experiment in a dark room illuminated only by LCD display and they were adapted to the environment illumination for at least 2 min before the recording session started. The display was properly surrounded by black foam to eliminate light reflection and interference from background texture.

Stimulus generation and experiment procedure were controlled by MATLAB (MathWorks) scripts written using Psychophysics and Eyelink Toolbox extensions [18], [19], [20].

### Procedure

Participants were seated and constrained by a chin rest to perform fixation and saccade task. A block was composed of 64 trials, of which 32 trials of each task were randomly interleaved. For both tasks, an adapt-test regimen was used and only one adaptation contrast was used in a single block. Block started with a 30 s block-adaptation, and followed by a 6 s trial-adaptation in each trial. Block and trial structure are illustrated in **Figure 1A**.

In achromatic blocks, achromatic grating were used as adaptation and test stimuli, whereas in chromatic blocks, chromatic grating were used. Adaptation grating and test grating had the same horizontal orientation. In saccade trials, participants were asked to fixate a central fixation cross (0.4°). After 400 ms, the cross disappeared and a target cross (0.4°) appeared 10° away on the right along the horizontal meridian, to which a saccade was made immediately. A one frame test grating patch (8.3 ms at 120 Hz refresh rate) was flashed about 190–230 ms (saccade latency for participants) after target onset, randomly centered 4° above or below the fixation cross. The saccade latency of each participant was determined in pilot experiment to ensure that test grating was presented in the range of 8.3 to 50 ms prior to the saccade onset (**Figure 1D**). In fixation trials, participants maintained fixation on the fixation cross, which was continuously displayed at the center of the monitor. Test grating was flashed after a fixation of 590–630 ms (sum of fixation duration and saccade latency in saccade trials; this duration is constant for the single participant but generally different from that of others), which was a comparable time after trial-adaptation with that of saccade trials.

After the test grating was presented, participants pressed keys on a gamepad (Microsoft SideWinder Game Controller) to indicate the perceived position of the test grating. The correctness of the response was recorded and fed into the QUEST core to produce a suggested value for the contrast of the next test grating. After the block was finished, estimates of fixation and saccade contrast threshold and their standard deviations were immediately given by QUEST algorithm, converging at a 75% correct performance. Blocks without a typical convergence curve (**Figure 1C**) of the test grating contrast would be abandoned and repeated. To maintain observers’ alertness on the task, a 5 min rest was allowed after each block. Three blocks (three adaptation contrasts for achromatic or chromatic condition) must be finished in one session. Chromatic and achromatic sessions were conducted on different days.

### Eye Movements

Eye movements were monitored by an infrared eye tracker (940 nm) Eyelink 1000 (SR research), sampling at 2 kHz. Saccade and fixation were evaluated by online parser with a saccade velocity criterion of 22°/s. The time of saccade onset, termination and test grating presentation were available immediately after the completion of each trial, as well as the validity of the participants’ performance. Trials, in which occasional blink or unqualified saccade (saccade end position was out of a 3°×3° window centered on target cross) happened, or test grating was not presented in the expected time range, would be discarded and repeated.

### Data Analysis

Contrast threshold and standard deviation were given by QUEST algorithm, online-recorded and sorted by a MATLAB working thread.

For simplicity in calculation, we used contrast threshold instead of contrast sensitivity, which is the inverse of contrast threshold. Since contrast threshold, as well as contrast sensitivity, is logarithmic measure in nature, a gain modulation (in decibel) which was commonly used as a measure of gain change or attenuation, was utilized to quantify the amplitude of saccadic suppression:
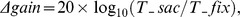



where *T_sac* and *T_fix* are contrast thresholds of saccade and fixation conditions at the same adaptation contrast. Obviously, the gain modulation defined here will give same value whether we use contrast threshold or contrast sensitivity as the original measure.

The significance of the main experiment operations was analyzed in SPSS with Repeated Measures ANOVA of a 2×2×3 (saccade/fixation, achromatic/chromatic, adaptation contrasts) within-subject design. Other tests would be stated where they were used.

## Results

**Figure 2:**
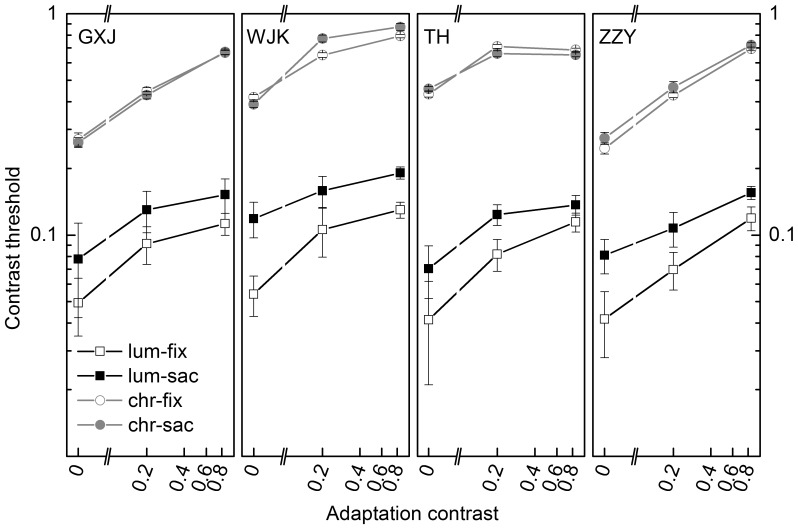
Contrast thresholds when observers were adapted by chromatic and achromatic grating. Fixation and saccade are abbreviated as fix. and sac., and chromatic and achromatic as chr. and achr. Three adaptation contrasts 0, 0.2 and 0.85 were used except for participant TH in his chromatic condition (for simplicity, not illustrated in the data panel). Error bar reports the standard error given by QUEST procedure. Each single line depicts the elevation of contrast threshold after contrast adaptation. For achromatic condition (black line), saccadic suppression is expressed by the distance between fixation (unfilled) and saccade (filled) threshold lines. For chromatic condition (gray line), the two lines almost overlap.

To assess the effect of adaptation operation, a proper combination of adaptation contrasts was used (see **Stimulus**) for all participants to avoid ceiling and floor effect (except for TH, a lower contrast combination was used in his chromatic adaptation blocks. For simplicity, this exception is not illustrated in **Figure 2**). Adaptation effect was examined by oral report and changes of the contrast threshold. In **Figure 2**, contrast thresholds in fixation and saccade conditions were both progressively raised along with the elevation of adaptation contrast, indicating that the adaptation operation was effective and the adaptation level of visual system was successfully manipulated as expected. The main effect of contrast adaptation was significant, F(1,3) = 84.2, p<.01. Pairwise comparisons further showed significant contrast adaptation in all conditions of achromatic adaptation [all p<.05]. However, in fixation and saccade conditions of chromatic adaptation, difference between adaptation contrast 0.2 and 0.85 was not significant [fixation, F(1,3) = 5.0, p>.05; saccade, F(1,3) = 4.7, p>.05], possibly caused by the data of observer TH and WJK, whose performance in chromatic condition showed slightly ceiling effect at greater adaptation contrasts.

**Figure 3:**
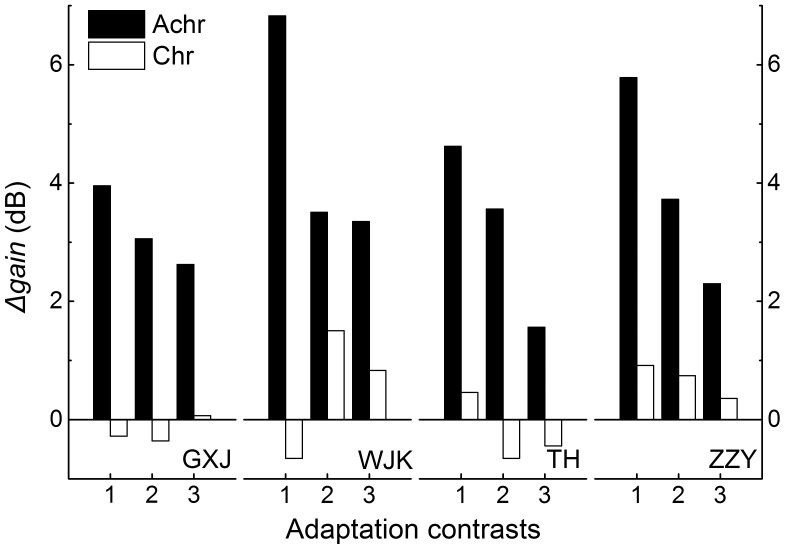
Saccadic suppression quantified with *Δgain.* Chromatic and achromatic are abbreviated as chr. and achr. Three adaptation contrasts are ordinal-indexed for illustration. *Δgain* shows a clearer and more robust decreasing tendency in achromatic condition (filled) than that in chromatic condition (unfilled) as contrast adaptation is intensified.

**Figure 4:**
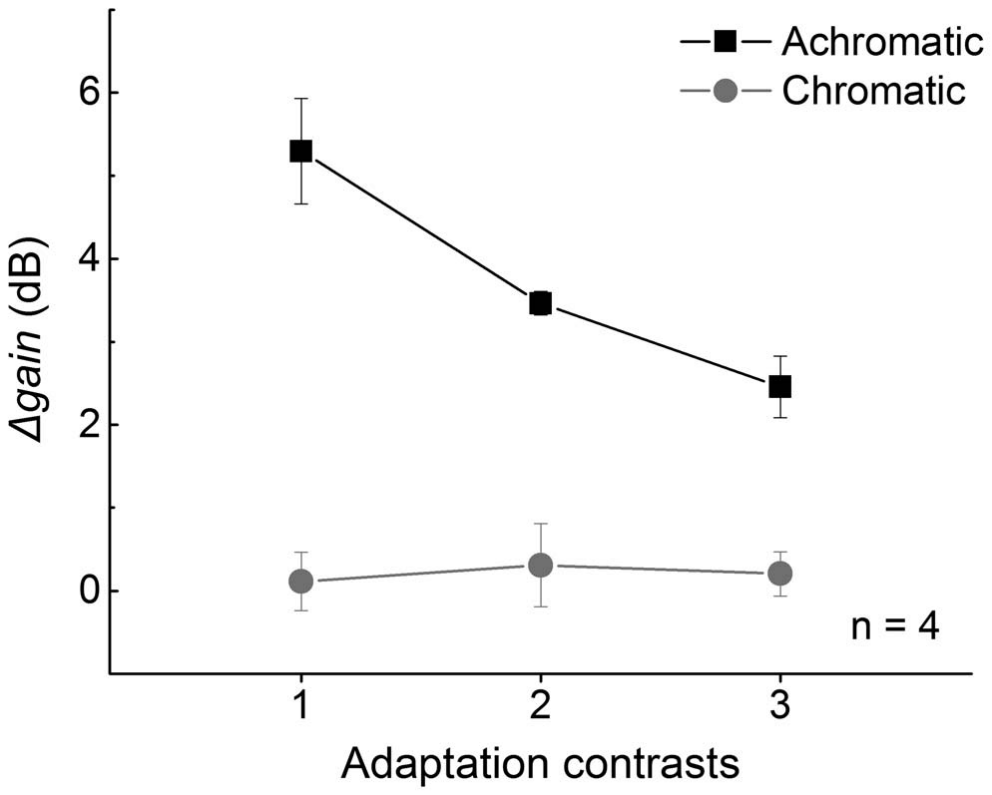
Average *Δgain* across participants. The same tendency as that in **Figure 3** is illustrated (achromatic condition, black square; chromatic condition, gray circle). Error bar shows the standard error of mean.

As the adaptation level was maintained at different steady states, contrast thresholds in saccade and fixation conditions were measured. We obtained the amplitude of saccadic suppression by comparing contrast thresholds of saccade and fixation. Significant and consistent saccadic suppression was observed in achromatic condition [F(1,3) = 136.6, p<.01], while in chromatic condition, no significant suppression was noticed [F(1,3) = .7, p>.05] (**Figure 2**). The discrepancy between achromatic and chromatic channel in saccadic suppression has been widely reported. In our experiment, chromatic condition was included as a control to justify the effect of achromatic condition and verify that the absence of saccadic suppression in chromatic condition would not be systematically altered by contrast adaptation. Saccadic suppression was then quantified with *Δgain* (see **Data analysis**). The variation of *Δgain*, plotted in **Figure 3**, illustrates how the amplitude of saccadic suppression changed when the gain adjustment mechanism was chromatically or achromatically adapted by greater contrasts. In achromatic condition, *Δgain* monotonically decreases as the adaptation level rises for all participants, showing that saccadic suppression is inversely correlated with the adaptation level [r(12) = −0.82, p<.001, Kendall’s Tau-b]. On the contrary, only a small amount of gain modulation is observed in chromatic condition. We have noticed a consistent decline from the data of participant ZZY, but no consistent dependence on the adaptation level emerges across participants. The salient disparity is explicitly demonstrated by group means in **Figure 4**.

## Discussion

In this paper, we psychophysically examined the relationship between contrast adaptation and saccadic suppression. Our result shows that in achromatic condition, saccadic suppression decreases as the contrast adaptation level of visual system increases, while in chromatic condition, saccadic suppression is not significant at all adaptation contrasts and no consistent tendency was induced by increasing adaptation contrast. The result is discussed hereafter.

### What does Contrast Adaptation Change?

We used block- and trial-adaptation in the adapt-test paradigm to manipulate contrast adaptation state like earlier adaptation studies [21], [22], [23]. The prominent function of contrast adaptation is to maximize perceptual power across prevailing contrasts. Psychophysical studies have found that contrast threshold was uniformly elevated by stronger contrast adaptation, although consistent enhancement of contrast discrimination sensitivity around adaptation contrast was not detected [23], [24], [25]. However, it was suggested that test conditions may be responsible for the contradictory results [23], [24], meanwhile studies showing enhanced contrast discrimination sensitivity did use similar stimulus arrangements to perform contrast adaptation operation as we did [25], [26]. Reports from single cell recording experiments also unveiled the neural mechanism of contrast adaptation by describing a wide variety of contrast-induced gain changes [22], [27], [28], [29]. As shown in **Figure 2**, fixation and saccade contrast thresholds were elevated as adaptation contrast increased, implying that contrast gain was changed by contrast adaptation operation so that fixation and saccade tasks were performed with the same baseline of contrast gain in a single block, i.e., contrast thresholds of saccade and fixation were tested based on the same population contrast response property. The difference between the two thresholds, i.e. saccadic suppression, could then be regarded as the influence posed on contrast gain by saccade. Thus the interaction between saccadic suppression and contrast adaptation can be interpreted like this: saccadic suppression is produced on the basis of different contrast gain baselines that have been modified by contrast adaptation.

### How is Saccadic Suppression Affected?

Contrast adaptation has not been used before to evaluate the involvement of contrast gain control in saccadic suppression. We observed negative correlation between contrast adaptation and saccadic suppression in achromatic condition. This can be interpreted in terms of contrast gain as follows: After adaptation, contrast response function (CRF) was shifted by ambient stimulus contrasts, accompanied by the change of contrast threshold. In our experiment the contrast threshold of fixation was elevated by greater adaptation, however, it grew slower as gain modulation was getting stronger **(** **Figure 2**, fixation contrast threshold), implying that the limited capacity of gain modulation was further restricted by greater contrast adaptation. Since saccade shared the identical gain with fixation, less room was left for saccadic modulation thereafter (i.e. saccadic suppression became smaller). Moreover, perceptually, greater suppression power should be devoted into lower contrast range since lower contrast information is dominant in natural environment [30], implying again why it is a negative, not positive correlation between contrast adaptation and saccadic suppression. Our results therefore indicate that saccadic suppression has a mechanism that can be mediated by contrast gain control.

Although our experiment gave direct evidence for the correlation between saccadic suppression and contrast gain control, we deliberately avoid further expanding details of the relationship for two reasons. First, contrast gain control mechanism in itself is a big concept which can be categorized by its temporal profile, influence on CRF and underlying physiological mechanism [31]. It was originally composed in electrophysiological studies [21], [22], making it difficult to be evaluated similarly at behavioral level. Second, saccadic suppression also includes contributions from many factors [32], visual masking, motion blur and corollary discharge, active or passive, retinal or non-retinal, as we have known. Without exquisite experiment design and robust techniques to identify components of saccadic suppression, it is not convincing to make direct connections between different contrast gain control mechanisms and saccadic suppression of diverse origins, if ever existed. For example, presenting brief test stimulus slightly before saccade onset was regarded as an acceptable estimation of active (or non-retinal) saccadic suppression, combining careful control of retinal smear and visual masking by designing a horizontal saccade across a parallel grating on a uniform gray background [1], [33]. Our experiment was conceived and conducted with the same stimulus arrangement. The objective of control measures, first, was to ensure that test grating patch was presented at the identical retinal position under fixation and saccade and in the time interval closest to the strongest suppression, and then, to conveniently test mainly active saccadic suppression. In sum, due to the limitation of psychophysical method and experiment design, our experiment is not qualified to elaborate the relationship between saccadic suppression and contrast gain control.

Combining paradigms of contrast adaptation and saccadic suppression, our study extends the knowledge of contrast gain control mechanism in saccadic suppression. However, we cannot conclude that saccadic suppression is achieved by contrast gain control, which requires detailed examinations along the visual pathway using electrophysiological method. Experimental and theoretical evidences are still needed to answer whether and how contrast gain control could be physiologically related to different contributions of saccadic suppression.
